# Oxidative stress alters mitochondrial homeostasis in isolated brain capillaries

**DOI:** 10.1186/s12987-024-00579-9

**Published:** 2024-10-15

**Authors:** Gopal V. Velmurugan, Hemendra J. Vekaria, Anika M.S. Hartz, Björn Bauer, W. Brad Hubbard

**Affiliations:** 1 Spinal Cord and Brain Injury Research Center, University of Kentucky, Lexington, USA; 2 Department of Neuroscience, University of Kentucky, Lexington, USA; 3 Sanders-Brown Center on Aging, University of Kentucky, Lexington, USA; 4 Department of Pharmacology and Nutritional Sciences, University of Kentucky, Lexington, USA; 5 Department of Physiology, University of Kentucky, Lexington, USA; 6 Department of Pharmaceutical Sciences, University of Kentucky, Lexington, USA; 7 Lexington Veterans’ Affairs Healthcare System, Lexington, USA

**Keywords:** Endothelial cells, Blood vessel, Mitochondria, Fission, Oxygen-glucose deprivation, Microvessels, Small vessel disease

## Abstract

**Background:**

Neurovascular deficits and blood-brain barrier (BBB) dysfunction are major hallmarks of brain trauma and neurodegenerative diseases. Oxidative stress is a prominent contributor to neurovascular unit (NVU) dysfunction and can propagate BBB disruption. Oxidative damage results in an imbalance of mitochondrial homeostasis, which can further drive functional impairment of brain capillaries. To this end, we developed a method to track mitochondrial-related changes after oxidative stress in the context of neurovascular pathophysiology as a critical endophenotype of neurodegenerative diseases.

**Methods:**

To study brain capillary-specific mitochondrial function and dynamics in response to oxidative stress, we developed an ex vivo model in which we used isolated brain capillaries from transgenic mice that express dendra2 green specifically in mitochondria (mtD2g). Isolated brain capillaries were incubated with 2,2’-azobis-2-methyl-propanimidamide dihydrochloride (AAPH) or hydrogen peroxide (H_2_O_2_) to induce oxidative stress through lipid peroxidation. Following the oxidative insult, mitochondrial bioenergetics were measured using the Seahorse XFe96 flux analyzer, and mitochondrial dynamics were measured using confocal microscopy with Imaris software.

**Results:**

We optimized brain capillary isolation with intact endothelial cell tight-junction and pericyte integrity. Further, we demonstrate consistency of the capillary isolation process and cellular enrichment of the isolated capillaries. Mitochondrial bioenergetics and morphology assessments were optimized in isolated brain capillaries. Finally, we found that oxidative stress significantly decreased mitochondrial respiration and altered mitochondrial morphology in brain capillaries, including mitochondrial volume and count.

**Conclusions:**

Following ex vivo isolation of brain capillaries, we confirmed the stability of mitochondrial parameters, demonstrating the feasibility of this newly developed platform. We also demonstrated that oxidative stress has profound effects on mitochondrial homeostasis in isolated brain capillaries. This novel method can be used to evaluate pharmacological interventions to target oxidative stress or mitochondrial dysfunction in cerebral small vessel disease and neurovascular pathophysiology as major players in neurodegenerative disease.

**Supplementary Information:**

The online version contains supplementary material available at 10.1186/s12987-024-00579-9.

## Background

Accumulating evidence from preclinical, postmortem, and epidemiological studies demonstrates a strong link between neurovascular dysfunction and cognitive impairment [1, 2]. Indeed, blood-brain barrier (BBB) damage and neurovascular deficits are major hallmarks of neurodegenerative diseases, such as Alzheimer’s disease (AD), vascular contributions to cognitive impairment and dementia (VCID), epilepsy, stroke, and traumatic brain injury (TBI) [1–6]. The underlying mechanisms of neurovascular pathophysiology are a strong therapeutic target for neurological diseases associated with neurovascular damage. Capillaries of the central nervous system (CNS) are specialized structures that restrict permeability through the BBB compared to endothelium in the periphery [7]. This unique property is achieved by tight junction proteins in brain capillary endothelial cells, which tightly seal the paracellular pathway and prevent unregulated passage of water-soluble molecules between blood and brain compartments [8]. The BBB maintains the ionic composition of the CNS extracellular fluid (ECF) and protects neurons and glial cells in brain parenchyma from plasma proteins [7]. A biomedical barrier is set up by transporters such as p-glycoprotein (P-gp) and breast cancer resistance protein (BCRP), as well as by metabolizing CYP 450 enzymes responsible for drug and fatty acid metabolism. Both efflux transporters and metabolizing enzymes are found in brain endothelial cells where they protect the brain from harmful substances and compounds [9, 10]. Overall, the specialized features in brain capillaries are thought to require additional energy supply compared to fenestrated or discontinuous capillaries in other organs. Indeed, there is increased mitochondrial content in brain capillary endothelial cells (8–11% of the cytoplasmic volume) compared to capillaries that are not derived from the BBB (2–5% of the cytoplasmic volume) [7, 11].

In general, mitochondrial activity plays a critical role in maintaining normal cellular function [12] and, in turn, mitochondrial homeostasis is crucial for endothelial cell function [13, 14]. Endothelial cells are shown to rely on mitochondria for important processes, such as calcium signaling and vasodilation [15]. Neurodegenerative diseases and acute brain injuries accumulate elevated levels of oxidative stress that can impact energy homeostasis. Excessive mitochondrial reactive oxygen species (ROS) and changes in mitochondrial dynamics in vascular endothelial cells result in vascular dysfunction [13]. Angiogenesis is a complex biological process that plays a critical role in metabolic supply by restoring blood flow, although this process is compromised in neurodegenerative diseases and brain trauma [16]. Mitochondrial dysfunction in capillaries correlates with impairment in angiogenic capacity [13]. Further, mitochondrial dysfunction and calcium overload have been implicated in vascular constriction and high blood pressure [17]. Increased blood pressure in humans causes capillary rarefaction and angiogenic capacity due to mitochondrial dysfunction [14].

Reports also demonstrate that microvascular oxygen delivery and brain capillary respiration are compromised with age [18, 19]. Indeed, cerebral vascular endothelial cells display decreased function in response to the mitochondrial ETC complex I inhibitor, rotenone [20]. Importantly, mitochondrial dysfunction and mitochondrial oxidative stress in brain endothelial cells can drive blood-brain barrier disruption [21]. Indeed, our group and others have shown that mitochondrial impairment is concurrent with BBB dysfunction in various neurogenerative diseases including mild TBI [5, 21, 22], and therapeutic targeting of mitochondrial-related oxidative stress and mitochondrial biogenesis improves behavioral outcomes after TBI [21, 23–25].

Although restoration of brain capillary health in stroke, TBI, and other neurodegenerative diseases should improve outcomes, there are limited models for drug screening specific to brain capillary oxidative stress and mitochondrial mechanisms. In this study, we hypothesized that oxidative stress would produce quantifiable changes in brain capillary mitochondrial function and dynamics in an ex vivo isolated brain capillary model.

## Methods

### Animal procedures

All studies performed were approved by the University of Kentucky IACUC, which is accredited by the Association for the Assessment and Accreditation for Laboratory Animal Care, International (AAALAC, International), and all experiments were performed per its guidelines. All animal experiments were compliant with the ARRIVE guidelines, and the experiments were carried out following the National Institutes of Health Guide for the Care and Use of Laboratory Animals (NIH Publications No. 8023, revised 2011). Initial capillary isolation and standardization experiments were performed using Sprague Dawley rats (Charles River) or WT C57/B6 mice (Jackson Labs) at 7–8 weeks old. Mice (B6;129 S-Gt (ROSA)26Sortm1.1 (CAG-COX8A/Dendra2) Dcc/J) expressing a global mitochondrial-specific version of Dendra2 green (mtD2g; homozygous) from Jackson Laboratories (Strain#: 018397) were utilized in other experiments. This strain was developed by crossing mtD2g floxed mice with Mesox2-Cre (germline delete) mice. In addition, we generated and used astrocyte-specific (Aldh1l1-CreER2:mtD2^f/f^) mitochondrial fluorescent reporter mice (Ast-mtD2). Both mtD2 flox (strain#:018385) and Aldh1l1-CreER2 (strain#:031008) mice were purchased from Jackson laboratories. All the mouse colonies were maintained at the Division of Laboratory Animal Resources at the University of Kentucky. Experiments were performed using male mtD2g mice at 12–16 weeks old with an average body weight of 25 g.

### Brain capillary isolation

**Fig. 1:**
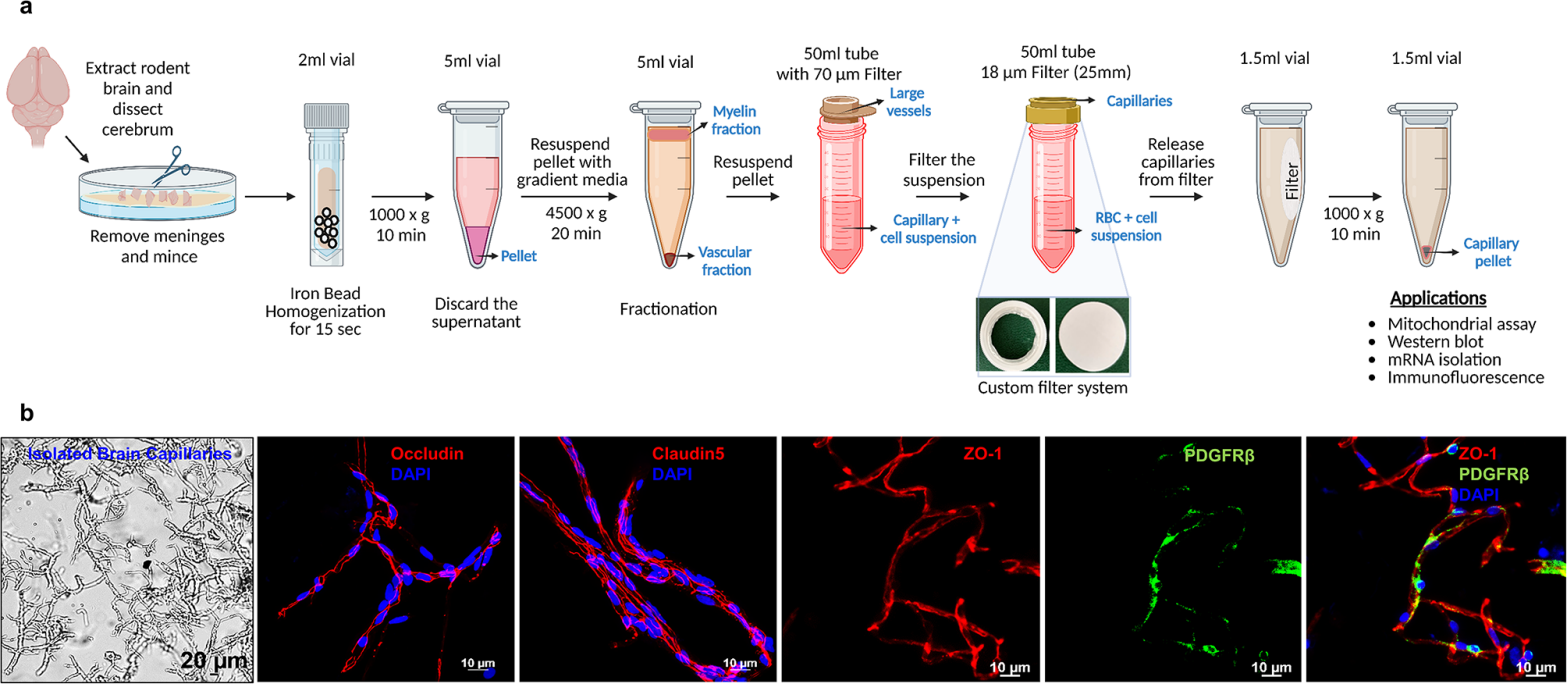
Efficient isolation method for brain capillaries with preserved cellular integrity. (**a**) Workflow of brain capillary isolation (subset shows modified filter holder; 25 mm). The last microcentrifuge tube shows a visible capillary pellet from a single mouse brain. (**b**) Representative micrographs of isolated brain capillaries. Bright-field image of isolated capillaries and isolated capillaries immuno-stained for tight junction protein and pericyte markers (occludin, claudin-5, ZO-1, and PDGFRβ)

After euthanasia with CO_2_, mouse brains were collected in ice-cold capillary isolation buffer (DPBS; Cat No: 14080055, Fisher Scientific with 5.5mM glucose, 1mM sodium pyruvate, and 1% BSA with pH 7.4). On ice, meninges, olfactory bulb and cerebellum were removed from a single mouse brain and the resulting cerebral hemispheres (~ 300–400 mg) were cut into small pieces using a razor blade and transferred into a 2 mL screw-cap tube (Sarstedt Inc Screw Cap Microtube, type H) preloaded with stainless beads (3.2 mm, 1.8 g, fisher scientific, NC0778455). 1 ml of ice-cold capillary isolation buffer was then pipetted into the tube before samples were homogenized in bead homogenizer (Biospec products) for 15 s. Tissue homogenate was transferred into a 5 ml tube. The screw cap tube and beads were rinsed with isolation buffer and transferred to the same 5 ml tube, and isolation buffer was added for final homogenate volume of 5 ml. Homogenate was vortexed quickly and centrifuged at 1000 × g for 10 min at 4 °C using a swinging bucket rotor. The supernatant was removed carefully, and 4 mL of lymphocyte separation medium (25-072-CV; Corning Life Sciences) was added to the pellet to separate the brain vessels from myelin and other brain cells by gradient centrifugation. The homogenate was vortexed for 30 s to obtain a homogenous suspension, which was then centrifuged at 4500 × g for 20 min at 4 °C using a swinging bucket rotor. The top myelin layer was removed along with the supernatant, and the inner sides of the tube were cleaned to remove leftover myelin debris using Kim Wipes or cotton buds. The pellet was resuspended in 1 ml of ice-cold isolation buffer, and the resulting homogenate was filtered through a 70 μm membrane filter (15-1070; Tisch Scientific) to remove larger vessels. The membrane was then washed with at least 10 ml of isolation buffer. The filtrate was then filtered through an 18 μm membrane (25 mm diameter) filter (ME17233; Tisch Scientific) assembled with a modified the filter holder (SF18128; Tisch Scientific), as shown in Fig. 1A. One 25 mm filter is sufficient to filter capillaries from one mouse cerebrum without clogging of the filter. At least 10 ml of wash buffer (DPBS; Cat No: 14080055, Fisher Scientific with 5.5mM glucose, 1mM sodium pyruvate with pH 7.4) was used to wash the membrane to remove RBCs, suspended brain cells and debris. Using tweezers, the 18 μm membrane filter with capillaries on the top was removed from the filter holder and placed inside the 1 ml microcentrifuge tube wall. Using a 1 ml pipette, all the capillaries were eluted from the membrane by flushing 1 ml of wash buffer 2-3X followed by quick vortexing. A drop of capillary elute was used to assess the quality and concentration of the capillaries under a bright field microscope. Final centrifugation was performed at 1000 × g, 10 min, 4 °C (swinging bucket) to pellet the isolated brain capillaries. Since capillaries are made up of a single cell layer of endothelial cells, we used endothelial culture media for subsequent ex vivo studies.

### AAPH and H2O2 treatment

Isolated capillaries were resuspended in endothelial cell culture medium (C-22011; PromoCell). Endothelial cell culture medium contains insulin-like growth gactor (Long R3 IGF, recombinant human) and vascular endothelial growth factor (VEGF). Each sample was divided equally into two parts in 1.5 ml microcentrifuge tubes and treated with 1mM AAPH or vehicle for 24 h or overnight in a 37 ℃ CO2 incubator. A hole was made in the microcentrifuge cap using a soldering iron gun for CO_2_ exchange. Following incubation, a small aliquot of capillary suspension (50 µl) was used for mitochondrial imaging in an 8-well chamber, and the rest of the capillary suspension was used for mitochondrial respiration analysis. Similarly, for H_2_O_2_ treatment (100 µM), capillaries were treated with H_2_O_2_ for 2 h followed by mitochondrial respiration and dynamics analysis.

### Mitochondrial respiration

Using a Seahorse XFe96 Flux Analyzer (Agilent Technologies, Palo Alto, CA, USA), the mitochondrial stress test was performed according to the manufacturer’s instructions. Briefly, capillaries from each group were centrifuged, and the pellet was resuspended in XF assay medium with substrates (10 mM glucose, 1 mM pyruvate, and 2 mM L-glutamine). Each sample was equally distributed as triplicates in XF microplates and centrifuged at 800 x g for 10 min to settle the capillaries at the bottom of the well. After 1 h of incubation at 37℃, the 96-well XF microplate was loaded into an XFe96 Flux analyzer to measure oxygen consumption rate (OCR). After three measurements of baseline OCR, respiratory chain inhibitors/uncouplers were sequentially injected into each well (1 µM Oligomycin, 4 µM FCCP, and 0.5 µM Rotenone/Antimycin A), and OCR was measured three times after each injection. At the end of the experiment, the XF microplate was centrifuged at 1000 g for 10 min, after removing the media, 10 µl RIPA buffer was added to the capillaries in each well, and freeze-thaw cycles were performed 3 times in dry ice before protein was quantified using a BCA kit (23225; Thermo Scientific). Using Wave software version 2.6.1 (Agilent Technologies, Santa Clara, CA, USA), OCR from various respiration states was calculated and normalized to protein concentration.

### Immunofluorescent staining

Isolated capillaries were loaded into an 8-well glass chamber slide to settle for 1 h at 25 ℃ before fixation with 10% neutral buffered formalin for 10 min. Mounted capillaries and brain sections were permeabilized and blocked (0.2% Triton X-100 in PBST, 1% BSA, and 10% normal horse serum) for 1 h at room temperature (RT). Then, incubated overnight at 4 °C with various primary antibodies including zona occludens-1 (ZO-1) (ZO1-1A12; Thermofisher), Occludin-1 (OC-3F10; Thermofisher), Claudin-5 (4C3C2; Thermofisher), platelet derived growth factor receptorβ (PDGFRβ) (3169 S; Cell Signaling), Isolectin B4, Biotinylated (B-1205-0.5; Vector laboratories), Aquaporin-4 (AQP4) (59678 S Cell Signaling;), TOM-20 (42406 S; Cell Signaling) and Glial fibrillary acidic protein (GFAP) (G3893; Sigma) were used at 1:250 dilution. Alexa flour 594 donkey anti-rabbit (A212207; Invitrogen), Alexa flour 488 donkey anti-mouse (A11001; Invitrogen), and Streptavidin, DyLight 649 (SA-5649-1; Vector laboratories) were used at 1:500 dilution as a secondary antibody at RT for 1 h. After washing, the samples were mounted on glass slides using a Vectashield HardSet Antifade Mounting Medium with DAPI (H-1500-10; Vector laboratories). Images were acquired using a Nikon confocal microscope with NIS-Elements version 5.30.05.

### Microscopy and plate reader

For mitochondrial dynamics analysis, Z-stack images were taken in 1 μm steps using a confocal microscope (Nikon A1R) in 100x oil immersion. Bright-field images were taken using Nikon widefield fluorescence (Nikon, Ti2) in 60X water objective. Protein concentration of capillaries were measured using BioTek Cytation 5 Cell Imaging Multimode Reader (Agilent Technologies, Palo Alto, CA, USA).

### Mitochondrial quantification in Imaris

The mitochondrial shape, size, and count were quantified as described previously [26]. Briefly, mtD2g green fluorescence from Z-stack images was quantified using Imaris (X64 9.6.1) using surface creation tool. A smoothed surface with background subtraction (local contrast) was used to separate the mitochondria from the background. A split-touching object was enabled to separate individual adjacent mitochondria. This template file was used to bulk-process all the images from different groups. Mitochondrial volumes (µm^3^) were frequently distributed with 0.5 bin width and 0–10 bin range (µm^3^). The percent distribution of mitochondrial volume was calculated by total number of mitochondria in each bin width percentage; (total number of mitochondria in each bin width/total number of mitochondria) *100. Mitochondrial volume (sum of total mitochondrial volume detected by Imaris surface detection tool) and count (count of mitochondrial segments detected by Imaris surface detection tool) were normalized to the total capillary area and expressed in 1000 µm^2^.

### Western blot

Western blot analysis capillary and whole brain lysates were prepared using RIPA buffer (150 mM NaCl, 1% Triton X-100, 0.5% sodium deoxycholate, 0.1% SDS, 50 mM Tris, pH 8.0), centrifuged at 16,100 x G for 30 min and total protein levels were estimated from supernatant using a BCA kit (23225, Thermofisher). Western blot samples were prepared using XT sample buffer (1610791, Biorad) with DTT and boiled at 95℃ for 10 min. Samples were resolved 4–12% BIS-TRIS gels (3450125, Biorad) under reducing conditions and transferred to a PVDF membrane. Probing against claudin-5 (1:1000; 4C3C2, Thermofisher), GFAP (G3893; Sigma), PDGFRβ (1: 1000; 3169 S; Cell signaling) and β-Actin (1:5000; 4970 S, Cell Signaling) was performed. Signals were detected using IRDy 68RD goat anti-mouse (1: 10,000; 926-68070, LI-COR biotechnology) and IRDye 800 CW goat anti-rabbit (1:10,000; 926-32211, LI-COR biotechnology) with LI-COR Odyssey DLx imager (LI-COR Biotechnology, Nebraska, USA). Capillary protein levels from western blot were quantified by densitometric analysis using ImageJ software, normalized to β-Actin and represented as fold changes compared to whole brain lysates.

### Statistics

Statistical analysis was performed using Graph Pad Prism (GraphPad Software, CA, USA). A significant difference among groups was defined as *p* < 0.05 for all analyses. The Shapiro-Wilk test was completed to ensure normality. As these criteria were met for all experimental data, parametric statistics were employed for all analyses. For parametric tests, normality tests were done after first ensuring the variable types are quantitative and continuous and not categorical or discrete. A two-tailed, unpaired t-test was used to examine the role of AAPH or H_2_O_2_ administration.

## Results

### Efficient capillary isolation method for capillary mitochondrial studies

Our methodology of capillary isolation (Fig. 1a) protocol features a streamlined process that preserves the quality and quantity of isolated brain capillaries. The ultimate goal of this method was to expedite the isolation process to preserve and measure capillary mitochondrial function and dynamics from a small amount of brain tissue, such as a mouse cerebrum. To achieve this goal, we standardized the existing protocol [27–29] with the following changes to increase efficiency: 1. Iron bead homogenization is performed quicker than dounce tissue homogenization 2. Brain homogenate preparation and vessel purification are carried out in microcentrifuge tubes, making the protocol suitable for rapid sample processing 3. A modified filter holder (Fig. S1) facilitates quick removal of the mesh filter with capillaries, eliminating the need to flush the filter from the opposite side to release the capillaries 4. Capillaries are eluted directly into microcentrifuge tubes, preventing the loss of capillaries that can occur when eluting into larger containers. Using this method, we obtained sufficient number of capillaries from a single mouse brain for subsequent mitochondrial bioenergetic studies, including technical replicates. Isolated brain capillaries displayed intact, tight junction proteins (ZO-1, Claudin-5, and Occludin-1) and pericytes (PDGFRβ) (Fig. 1b).

**Fig. 2:**
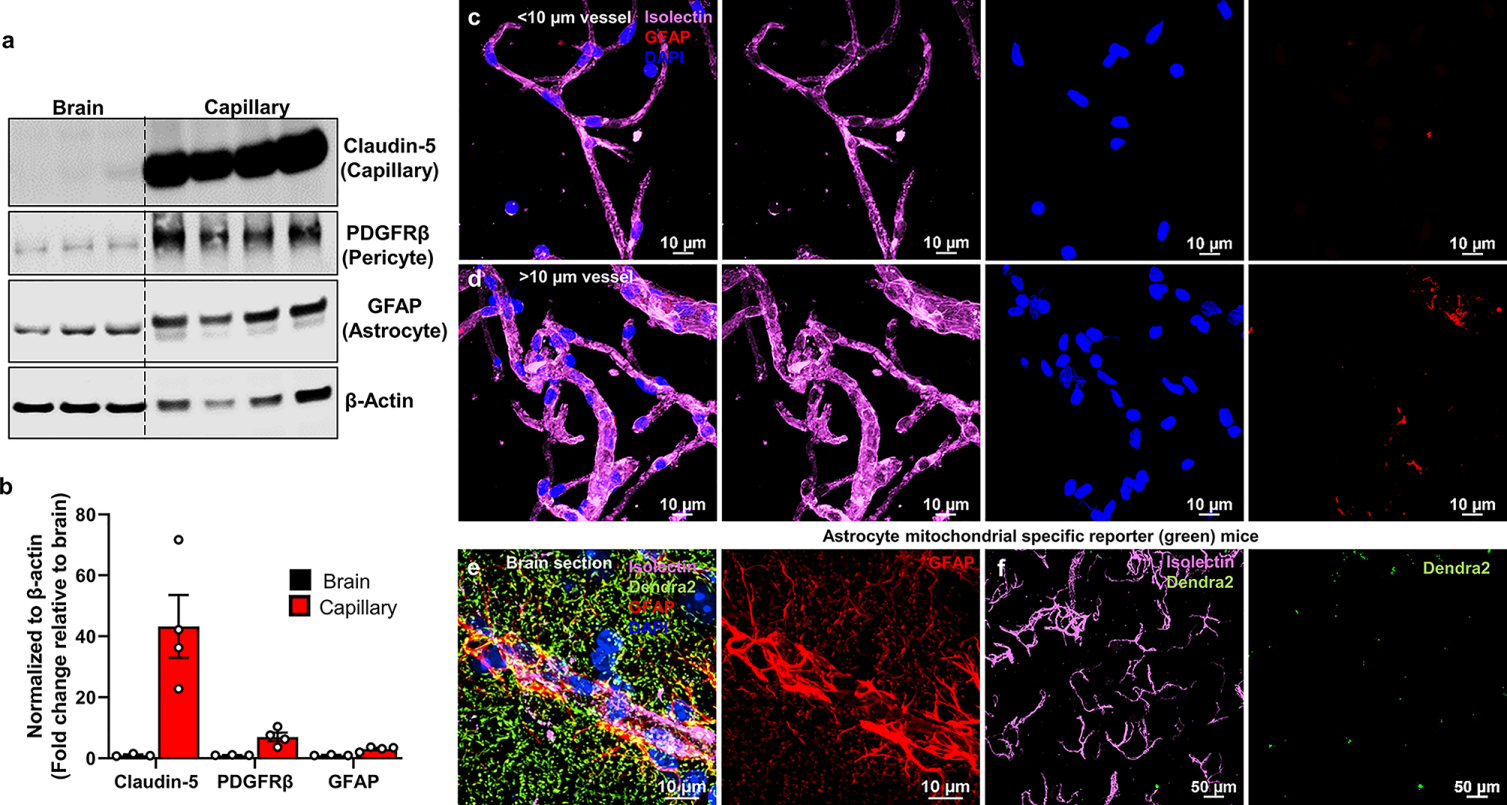
Purity and reproducibility of isolated brain capillaries. (**a**) Western blot micrograph from isolated brain capillaries (*n* = 4 mice) and whole brain lysates (*n* = 3 mice) for endothelial cell tight junction protein, claudin-5, pericyte marker PDGFRβ and astrocyte marker GFAP. (**b**) The bar graph represents normalized protein quantified from isolated brain capillary lysates compared to brain lysates. (**c**, **d**) Representative confocal micrographs from isolated capillaries were stained with astrocyte marker (GFAP; red), endothelial marker (isolectin; pink) and nucleus (DAPI; blue). Microvessels larger than 10 microns in diameter show some remnants of astrocyte GFAP (red) staining. (**e**) Representative micrographs from Ast-mtD2 brain section shows astrocyte specific mtD2 (green) expression. Also, astrocytic GFAP (red) expression around the capillary (pink) and in the brain matrix. (**f**) Micrograph of isolated brain capillary preparation (pink) from Ast-mtD2 mouse model

To evaluate the purity of our isolation, western blot was performed on the isolated capillaries and whole brain lysates for tight junction protein, claudin-5, the pericyte marker, PDGFRβ, and the astrocyte marker, GFAP (Fig. 2a). As expected, protein quantification showed approximately 40-fold and 7-fold higher expression of claudin-5 and PDGFRβ, respectively, in isolated capillaries compared to brain lysates (Fig. 2b). We found consistent elevated claudin-5 expression across samples from isolated brain capillaries compared to whole brain lysates, confirming the purity and reproducibility of our capillary isolation method. GFAP expression was also ~ 3-fold higher in isolated capillaries than in brain lysates (Fig. 2b), which may be due to increased GFAP expression in astrocytes surrounding the capillaries, compared to GFAP expression within the brain parenchyma (Fig. 2e). To assess the potential contribution of astrocytes and their mitochondria to brain capillary mitochondrial bioenergetics, we immunostained brain capillaries and brain sections from WT and Ast-mtD2 (astrocyte-specific mitochondrial labeled) mice using cell-specific markers (Fig. 2c-f). Over 95% of the capillaries isolated from WT mouse brains were GFAP-negative (Fig. 2c), with larger microvessels (> 10 μm in diameter) more likely to contain remnants of GFAP + astrocytes (Fig. 2d). Immunofluorescence images from Ast-mtD2 brain sections revealed that brain capillaries are closely associated with astrocytes and thereby astrocytic mitochondria (green) (Fig. 2e). However, isolated capillaries from these mice contained very few astrocytic mitochondria (Fig. 2f). Therefore, the contribution of astrocytic mitochondria to brain capillary mitochondrial bioenergetics is minimal.

### Feasibility of extended ex vivo incubation and mitochondrial outcomes of isolated brain capillaries

**Fig. 3:**
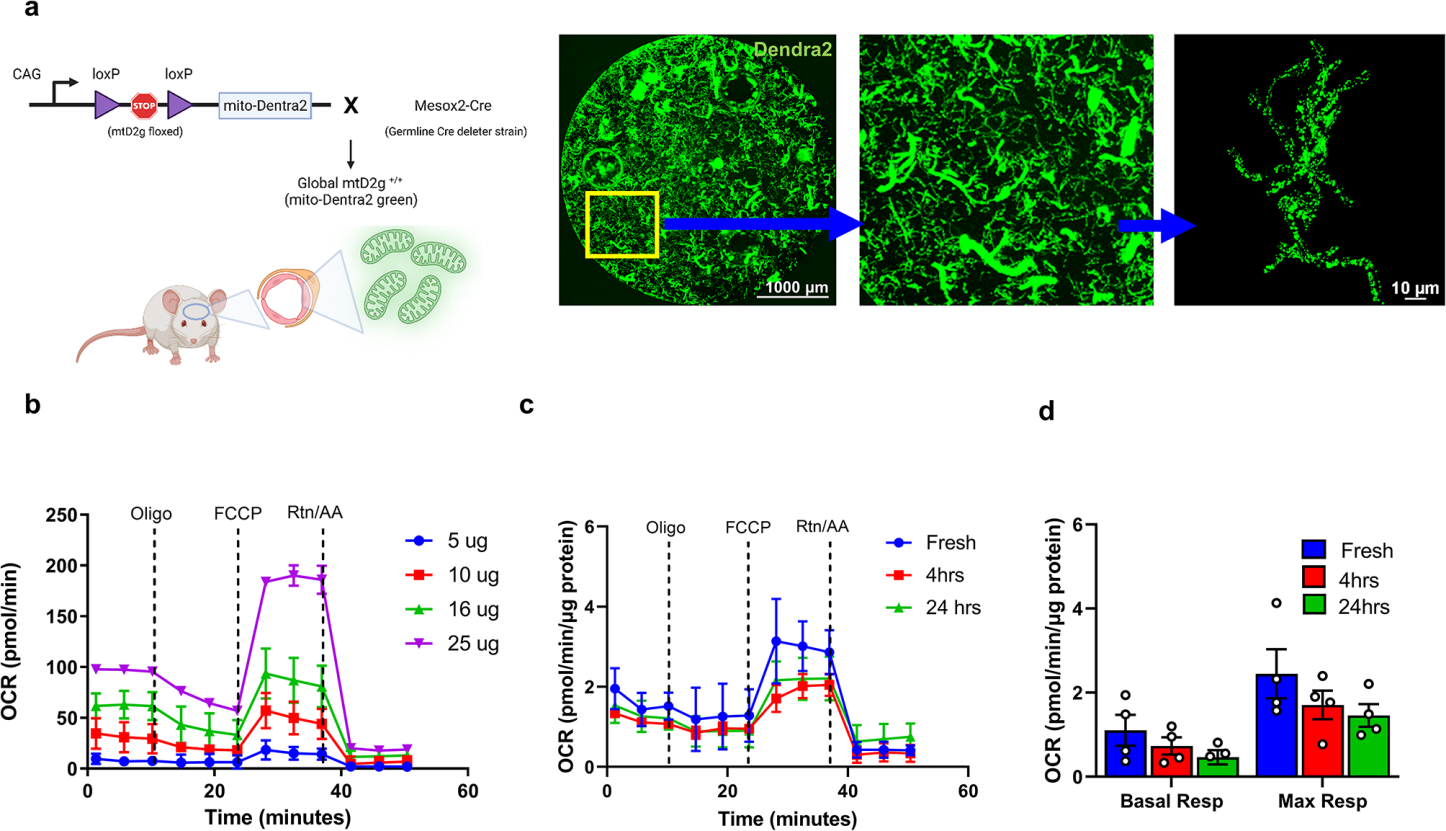
Feasibility of ex vivo incubation of isolated brain capillaries. (**a**) Schematic showing the generation of the mtD2g mouse model used in this study and representative micrograph of isolated mtD2g capillaries in a 96-well flux plate. (**b**) Representative traces of mitochondrial stress test (MST) using respiratory chain inhibitors (oligomycin, FCCP, and rotenone + Antimycin A) from different concentration of freshly isolated brain capillaries. (**c**) MST traces from fresh, 4 h and 24 h incubation of ex vivo mtD2g mice brain capillaries. (**d**) The bar graph represents the summary of basal and maximum oxygen consumption rate (OCR) in MST from fresh, 4 h and 24 h incubation ex vivo. OCR was normalized to capillary protein (*n* = 4 technical replicates)

**Fig. 4:**
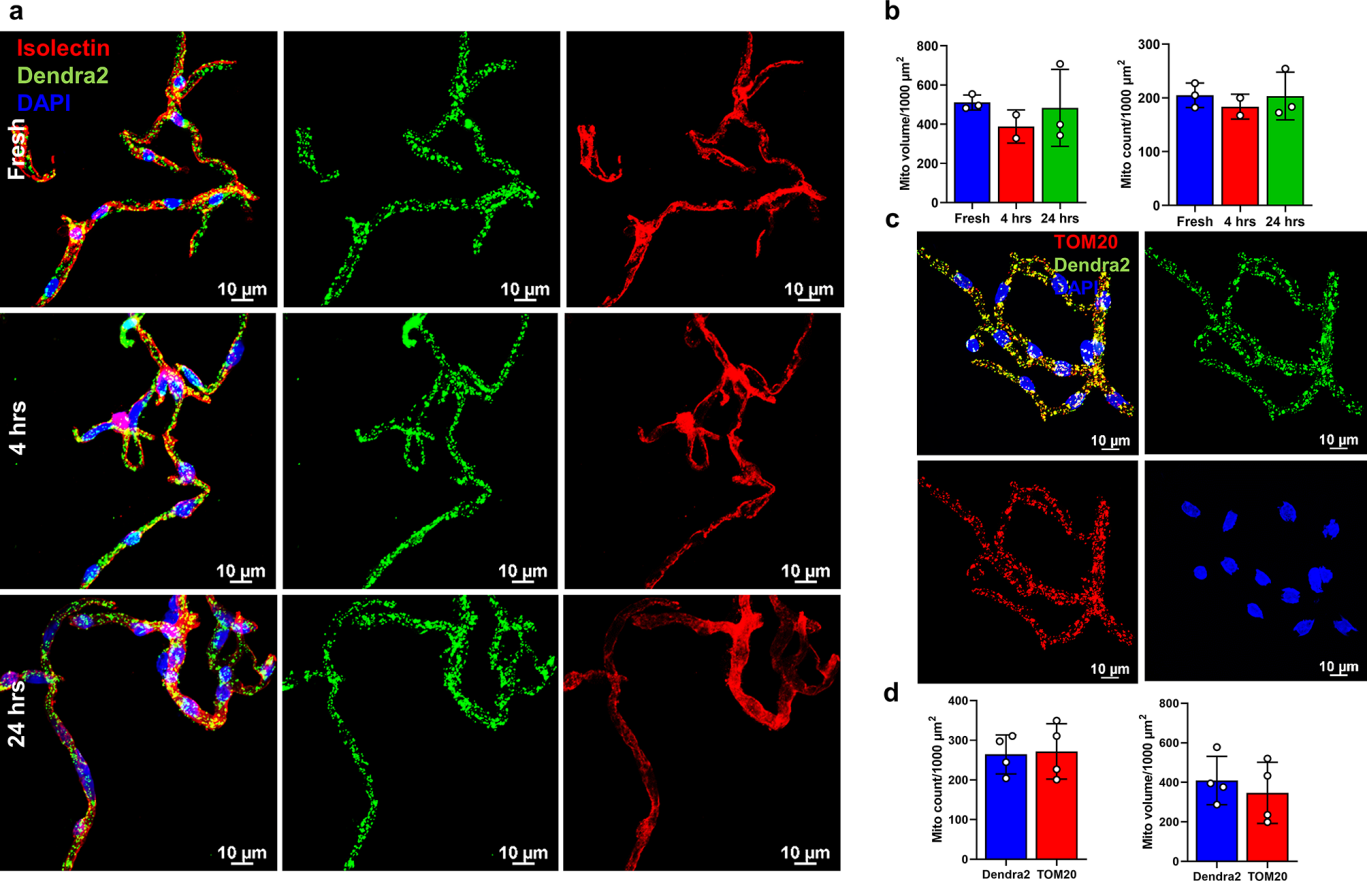
Mitochondrial dynamics assessment in isolated brain capillaries. Mitochondrial dynamics was analyzed in Imaris with a surface detection tool using confocal z-stack images. (**a**) Representative micrographs showing isolated capillaries (isolectin (red) and mtD2 (green)) from fresh, 4 h and 24 h ex vivo incubation. (**b**) Bar graph of mitochondrial volume and mitochondrial count in 1000 µm^2^ capillary surface area. (**c**) Representative confocal micrographs images from mtD2g capillaries immunostained with TOM-20 (red). (**d**) Bar graphs of mitochondrial volume and mitochondrial count in 1000 µm^2^ capillary surface area. Experiments were performed using isolated brain capillaries from mtD2g mouse (*n* = 4 capillaries randomly imaged). Data are represented as mean ± SD

To test the stability and feasibility of measuring mitochondrial function after the isolation process, we used isolated brain capillaries from globally expressing mtD2g mice (Fig. 3a). Capillary mitochondrial function was tested with various levels of isolated brain capillary protein by running a mitochondrial stress test (MST) in the Seahorse XFe96 flux analyzer and a linear response was observed between protein level and oxygen consumption rate (Fig. 3b). This allows a range of capillary protein levels to be used for bioenergetic analysis, which is significant as protein is not measured before the mitochondrial respiration assay. Isolated brain capillaries were incubated for 4 h and 24 h ex vivo in endothelial culture media and compared with freshly isolated capillaries for mitochondrial function and dynamics. Our results show no statistical difference in basal and maximal mitochondrial respiration (Fig. 3c, d) or mitochondrial dynamics, including mitochondrial volume and count, (Fig. 4a, b) between freshly-isolated, 4 h incubated, 24 h incubated brain capillaries. This demonstrates that functional mitochondrial activity and network is sustained in ex vivo isolated brain capillaries for at least 24 h. To demonstrate the versatility of this method, we used capillaries from transgenic mtD2g animals, immunostained with TOM20, and quantified mitochondrial dynamics using Imaris for both Dendra2 and TOM20 signals separately (Fig. 4c, d). The results indicate that TOM-20 staining can be utilized in WT animals to measure mitochondrial dynamics, in addition to the endogenous labeling that is provided by mtD2 transgenic animals.

### Dysregulation of mitochondrial function and dynamics following oxidative stress

**Fig. 5:**
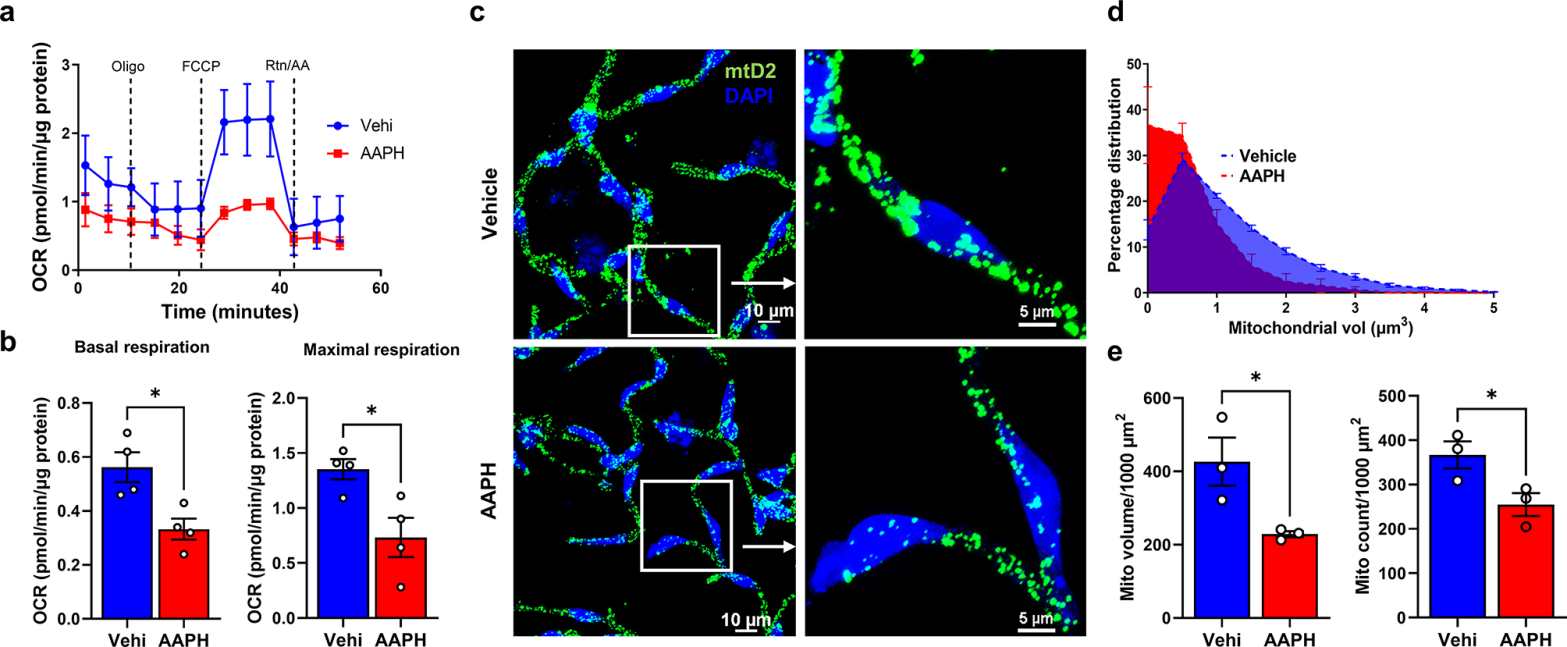
Mitochondrial function and dynamics from AAPH-treated mtD2g brain capillaries. (**a**) Traces from MST for vehicle and AAPH-treated mtD2g capillaries after 24 h ex vivo treatment. (**b**) The bar graph represents the summary of basal and maximum OCRs during the MST from vehicle and AAPH-treated mtD2g capillaries. OCR was normalized to capillary protein (*n* = 4 mice/group). (**c**) Representative confocal micrographs from mtD2g capillaries treated with AAPH or vehicle for 24 h. (**d**) The histogram summarizes the percent mitochondrial volume distribution from vehicle and AAPH-treated mtD2 capillaries (*n* = 3 mice/group). (**e**) Bar graphs of mitochondrial volume and mitochondrial count in 1000 µm^2^ capillary surface area. All the experiments were performed using isolated brain capillaries from homozygous global mtD2g mice. Data are represented as mean ± SEM. *p* ≤ 0.05 * using the unpaired t-test (**b** and **e**)

Our previous study established that AAPH-induced oxidative stress significantly changes mitochondrial function [26]. To test the ex vivo brain capillary mitochondrial function response to an oxidative insult, we treated isolated brain capillaries from mtD2g mice either with vehicle or AAPH (1mM) for 24 h and analyzed bioenergetics using Seahorse XFe96 Analyzer. Our results show that AAPH treatment decreased the mitochondrial respiration in brain capillaries compared to vehicle treatment (Fig. 5a). Summary data from mice (*n* = 4 mice/group) reveals that basal and maximal OCR, normalized to protein levels, was significantly decreased by oxidative stress compared to vehicle treatment (Fig. 5b). To assess if a change in capillary mitochondrial function consistently affects capillary mitochondrial dynamics as we observed previously [26], we used a new cohort of mice to generate isolated capillaries for confocal imaging (Fig. 5c) and performed mitochondrial dynamics analysis in Imaris. A histogram of mitochondrial volume percentage distribution (Fig. 5d) demonstrates that AAPH-induced oxidative stress significantly increased populations of mitochondria with lower volume, implicating a mitochondrial fission phenotype. Consistent with mitochondrial function, AAPH treatment also significantly decreased mitochondrial volume and count (Fig. 5e) compared to vehicle treatment.

**Fig. 6:**
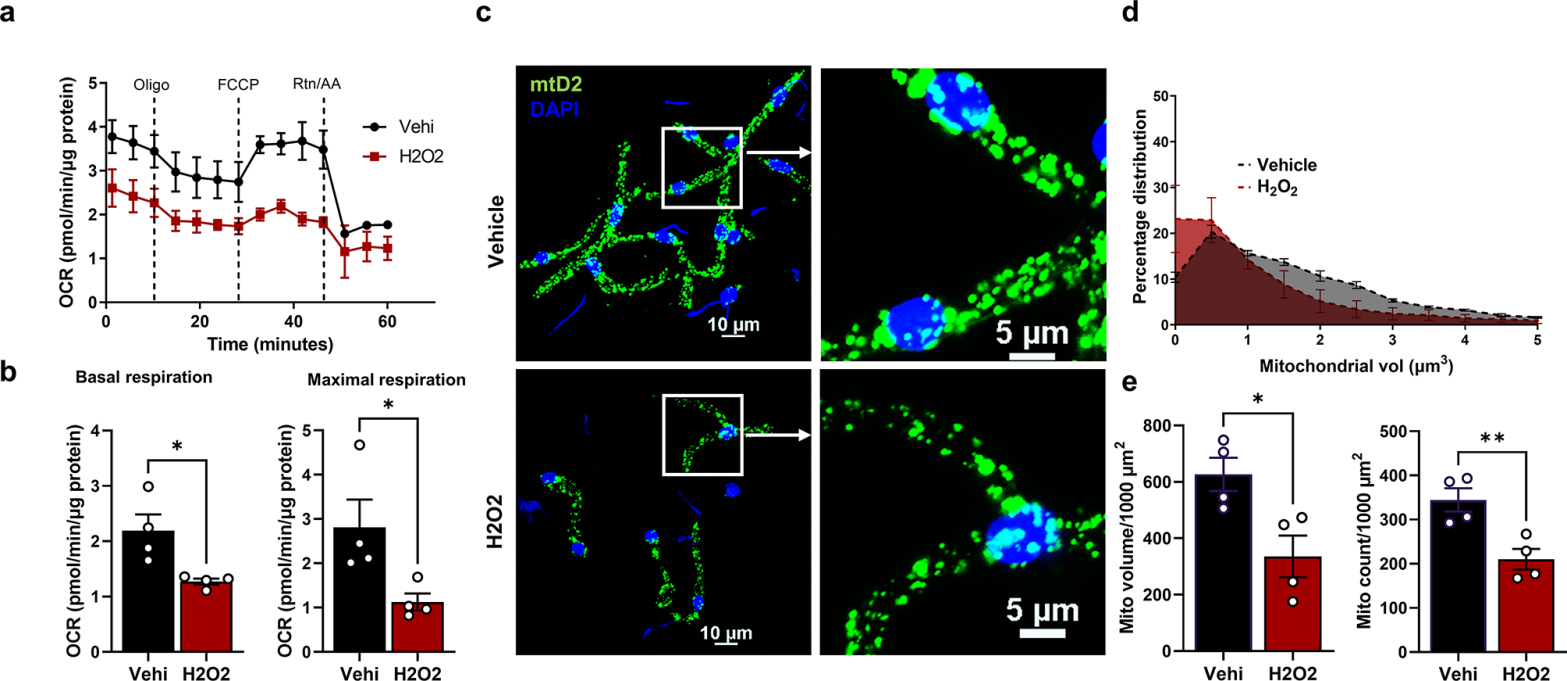
Mitochondrial function and dynamics from H _2_O_2_-treated mtD2g brain capillaries. (**a**) Traces from mitochondrial stress test (MST) for vehicle and H_2_O_2_ treated mtD2g capillaries after 2 h ex vivo treatment. (**b**) The bar graph represents the summary of basal and maximum OCRs during the MST from vehicle and H_2_O_2_-treated mtD2g capillaries. OCR normalized to capillary protein (*n* = 4 mice/group). (**c**) Representative confocal micrographs from mtD2g capillaries treated with H_2_O_2_ or vehicle for 2 h. (**d**) The histogram summarizes the percent mitochondrial volume distribution from vehicle and H_2_O_2_-treated mtD2 capillaries (*n* = 4 mice/group). (**e**) Bar graphs of mitochondrial volume and mitochondrial count in 1000 µm^2^ capillary surface area. All the experiments were performed using isolated brain capillaries from homozygous global mtD2g mice. Data are represented as mean ± SEM. *p* ≤ 0.05 *; *p* ≤ 0.01 ** using the unpaired t-test (**b** and **e**)

To generalize the response to oxidative stress, we also used H_2_O_2_ to induce oxidative stress in capillaries and assessed cellular bioenergetics. Similiar to AAPH experiment, H_2_O_2_ (100 μm for 2 h) treatment also significantly decreased basal and maximal mitochondrial respiration in brain capillaries (Fig. 6a, b). H_2_O_2_ also induced a mitochondrial fission phenotype (Fig. 6c, d) with decreased mitochondrial volume and count compared to vehicle treatment (Fig. 6e).

In addition to oxidative stress, other pathological conditions like oxygen-glucose deprivation (OGD)/reperfusion contribute to capillary mitochondrial dysfunction. We treated brain capillaries with OGD-reperfusion and normoxic conditions. We show a decrease maximum mitochondrial respiration (Fig. S2c) but not at basal respiration (Fig. S2b) for OGD-reperfusion groups compared to normoxia. We did not see any significant change in OGD-reperfusion for mitochondrial dynamics compared to normoxic conditions (Fig. S2 d, e, f)).

## Discussion

The neurovascular unit (NVU) consists of neurons, astrocytes, and blood vessels that work together to maintain brain health and function [8, 9]. This interconnected system ensures the delivery of nutrients and oxygen to the brain while regulating cerebral blood flow. Understanding the interplay between neurons, astrocytes, and capillaries is crucial for comprehending various neurological disorders. Mitochondrial dysfunction in neurons and astrocytes has garnered significant attention in research related to neurodegenerative diseases and TBI [30–32]. Mitochondria are vital organelles responsible for energy production and play a crucial role in maintaining cellular function, particularly in energy-demanding organs like the brain. Despite the acknowledged importance of mitochondrial dysfunction in neurons and astrocytes in these conditions, studies on brain capillary mitochondrial function are relatively scarce. As part of the NVU, capillaries play a pivotal role in cerebral blood flow regulation and nutrient/oxygen delivery to brain tissue. However, research focusing on their mitochondrial function is limited, potentially due to methodological challenges or the lack of established techniques to study these specific brain microvascular components. Novel imaging techniques, molecular probes, and more refined isolation methods pave the way for a better understanding of the role of capillary mitochondria in brain health and disease. Studying capillary mitochondrial function could offer new insights into the mechanisms underlying neurological disorders and possibly identify novel therapeutic targets. Addressing this knowledge gap could have significant implications for developing targeted interventions and treatments for various neurological conditions.

To study mitochondrial function in brain capillaries, we showcase an isolation method that results in sufficient quantity and quality of brain capillaries for downstream assays. Isolation of brain capillaries has been demonstrated previously [27, 28, 33]. This study was designed to achieve efficient, rapid isolation of brain capillaries while preserving BBB barrier integrity. Compared to previously published results [28], our isolation method demonstrates that capillaries are mostly (more than 95%) devoid of astrocyte marker GFAP by immunofluorescence analysis. Additionally, we observed approximately 40-fold increase in claudin-5 expression in capillary isolates relative to brain lysates, as shown by western blot analysis, underscoring the purity and cellular enrichment of the isolated capillaries. While GFAP expression in capillary lysates was approximately 3-fold higher than in brain lysates, this did not result in a significant contribution of astrocytic mitochondria as represented by minimal astrocytic mitochondrial presence in isolated capillaries, as determined by astrocytic-specific mitochondrial labeling in Ast-mtD2 mouse model, which revealed. It is plausible that the small amount of astrocyte remnants in the capillary preparation may account for the elevated GFAP protein levels in the capillary lysates compared to whole brain lysates. Consequently, our results strongly indicate that brain capillary mitochondrial function is driven by mitochondria from endothelial cells and pericytes, which are critical to capillary architecture, and not by astrocytic mitochondria.

In this study, we also tested the feasibility of ex vivo incubation of capillaries for up to 24 h. Since capillaries are composed of a single endothelial cell layer, we used endothelial cell culture medium to standardize ex vivo culture conditions. Surprisingly, we found that brain capillaries could maintain similar mitochondrial function even after 24 h ex vivo incubation compared to freshly isolated capillaries. This finding allows overnight treatment with chemical and therapeutic manipulations to target brain capillary function. Screening of therapeutic compounds and dose-response studies to rescue capillary mitochondrial function can be performed with this methodology.

Changes in mitochondrial dynamics, such as mitochondrial fission, affect mitochondrial function [26]. In this study, we found that lipid peroxidation increased mitochondrial fission phenotype in isolated brain capillaries with decreased brain capillary mitochondrial bioenergetics. Mitochondrial dyshomeostasis was found following overnight AAPH incubation or acute H_2_O_2_ incubation, demonstrating the generalizability of oxidative stress on mitochondrial outcomes in the context of brain capillaries. While we showcase the novelty of tracking endogenous mitochondrial fluorescent labeling as an index of mitochondrial morphology and dynamics, we also show that this can be achieved using TOM20 staining in brain capillaries isolated from WT animals.

## Conclusion

In summary, this efficient brain capillary isolation method provides a novel platform for studying capillary mitochondrial homeostasis ex vivo, particularly in the context of oxidative stress and neurodegenerative diseases. This robust brain capillary isolation method can be utilized for further research and drug screening related to mitochondrial and oxidative mechanisms in cerebral small vessels in the context of neurodegenerative diseases and brain trauma.

## Electronic supplementary material

Below is the link to the electronic supplementary material.

Supplementary Material 1

Supplementary Material 2

## Data Availability

The datasets used and/or analyzed during the current study are available from the corresponding author upon reasonable request.

## Abbreviations

AAPH: 2,2’-azobis-2-methyl-propanimidamide dihydrochloride
AD: Alzheimer’s disease
NVU: Neurovascular unit
AQP4: Aquaporin-4
BCRP: Breast cancer resistance protein
BBB: Blood-brain barrier
mtD2g: Dendra2 green specifically in mitochondria
VCID: Vascular contributions to cognitive impairment & dementia
CNS: Central nervous system
ECF: Extracellular fluid
P-gp: p-glycoprotein
ROS: Reactive oxygen species
TBI: Traumatic brain injury
ZO-1: Zona occludens-1
GFAP: Glial fibrillary acidic protein
OCR: Measure oxygen consumption rate
